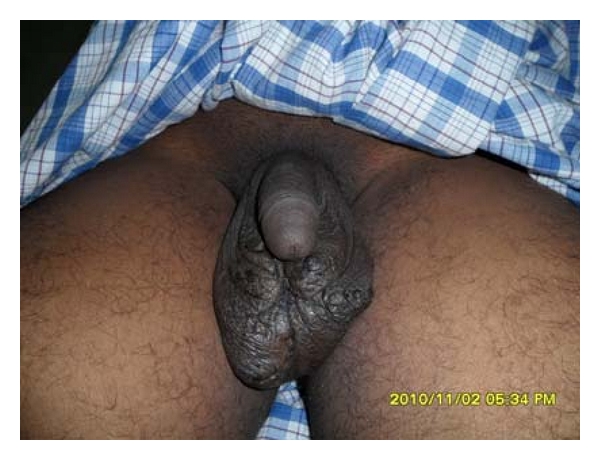# Atypical Steatocystoma Multiplex with Calcification

**DOI:** 10.5402/2011/381901

**Published:** 2011-07-12

**Authors:** Muhammad Hasibur Rahman, Muhammad Saiful Islam, Nazma Parvin Ansari

**Affiliations:** ^1^Cosmoderma Skin Cure Center 96/G, Nirmalabas, Sheora Road, Mymensingh 2200, Bangladesh; ^2^Department of Orthopedics, Community Based Medical College, Mymensingh 2200, Bangladesh; ^3^Department of Pathology, Community Based Medical College, Mymensingh 2200, Bangladesh

## Abstract

A 60-year-old male reported to us with an atypical case of giant steatocystoma multiplex in the scrotum with calcification. There was no family history of similar lesions. Yellowish, creamy material was expressed from a nodule during punch biopsy. The diagnosis was based on clinical as well as histological findings. Successful surgical excision was done to cure the case without any complications.

## 1. Introduction

Steatocystoma multiplex occurs as numerous, epithelial-lined, sebum-filled dermal cysts with characteristic sebaceous glands in the cyst walls [1]. Usually it begins in adolescence or early adult life. It may be inherited as an autosomal dominant trait, but most cases have no family history [2]. Both sexes are affected equally. Clinically, it is characterized by multiple small soft, movable, yellowish to skin-colored dermal cystic papules and nodules, varying from a few to 20 mm or more in size [3]. Overlying epidermis, it usually remains normal with no central punctum [4]. The trunk (with the presternal region as the site of election) and proximal extremities are involved, but lesions may appear anywhere, including the scrotum. Usually, the lesions are asymptomatic, but some lesions may become inflamed, suppurate and heal with scarring [3]. When inflammation of the ruptured cysts takes place and is extensive, it can produce the so-called steatocystoma multiplex suppurate, which mimics acne conglobata [2]. Occurrence of a solitary lesion is called steatocystoma simplex, which has no hereditary tendency [3]. The condition has been given, variety of names including steatocystomatosis, sebocystomatosis and epidermal polycystic disease [5]. Instances of lesions of large size grouped in large quantities in one region of the body are rare. Similar lesions with extensive calcification seem to be extremely rare. We are presenting here a case report of steatocystoma multiplex with extensive calcification and firm adhesion to the scrotum.

## 2. Case Report

**Figure 1:**
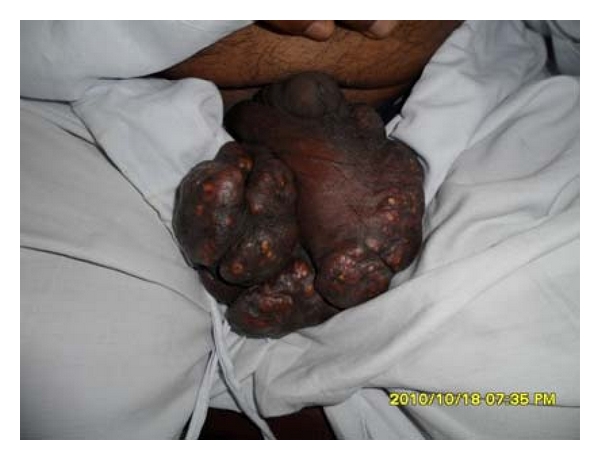


A 60-year-old male presented to us with asymptomatic multiple cystic papule and nodules of various size and shape in group with firm adhesion on the scrotum for a period of 15 years. To start with the investigation we noticed a small, firm, elevated lesion on the scrotum which remained stationary for 10 years. With the passage of time, and since the past year, the lesion increased in size and number. Large-sized nodules grouped in large quantities in scrotum are rarely found. Similar lesions with extensive calcification with firm adhesion seem to be extremely rare.  Figure 1 shows atypical steatocystoma multiplex with calcification as first presentation to us. Our investigation revealed that there was no history of similar skin lesions in other members of the family, and there was no association with any other cutaneous disorders.

On examination, it was evidenced that there were multiple large- to very large-sized, discrete, mobile, firm, yellowish to skin-colored, dermal cystic papules and nodules of various atypical shape, with adhesion to the skin distributed only in scrotum. Pricking the lesion expressed oily fluid from some of the lesions. There were no signs of inflammation in any of the lesions. Other systems were normal, and routine laboratory examination revealed no abnormality.

Skin biopsy of the cystic nodule revealed histologic features suggestive of steatocystoma multiplex. The cyst was located in the dermis with a folded cyst wall. The cyst wall had a basal layer in palisade arrangement of the cells towards the cavity. There were few flattened sebaceous gland lobules close to the cyst wall.

**Figure 2:**
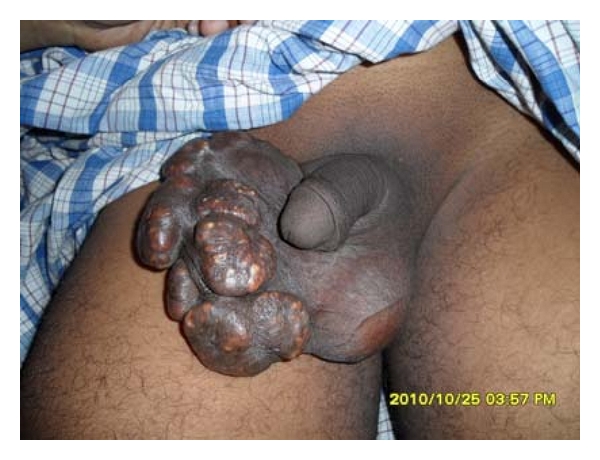


The lesions were surgically excised after giving spinal anesthesia to the patient.  Figure 2 shows the case before doing surgery and Figure 3 shows the case 10 days after surgery. Long-term followup revealed no recurrence or any other complications.

## 3. Discussion

 Steatoma or sebaceous cyst is due to an overproduction of sebum by one or more sebaceous glands while the usual outlet is closed. When this collection remains in situ for years, the peripheral pressure destroys the epithelial lining of the cyst, and the sebum may undergo calcareous degeneration.

Steatocystoma multiplex is an uncommon disorder that is characterized by multiple soft, movable, small, cystic nodules. The diameter of lesions typically varies from 0.2*∼*2 cm, but the nodules may be larger. The overlying epidermis is usually normal with no central punctum present. The lesions are typically located on the upper trunk, neck, axillae, scrotum, and proximal extremities. A few isolated steatomas scattered in various parts of the body are of frequent occurrence. Occasionally, one sees large numbers of the pinhead-sized or the pea-sized lesions. Although there are reports of involvement of various sites, such as scalp, forehead, chest, trunk, groin, axilla, scrotum, and so forth, an increase in size and number with extensive calcification and adhesion tightly to the scrotum for many years are unlikely and an asymptomatic presentation. 

The colour of the lesions varies from yellowish to skin colour and this variation has been attributed to the depth of the lesions—the superficial lesions being yellowish, and the deeper lesions skin coloured [2]. In our case, the lesions exhibited both the colors. 

The causative factors of steatocystoma multiplex still remaine unclear, yet Setoyama et al. [6] suggest that trauma, infection, or immunological events might be responsible. A few authors have reported that steatocystoma multiplex could be associated with ichthyosis and koilonychia, pachyonychia congenita, acrokeratosis verruciformis of Hopf, hypertrophic lichen planus, hypohidrosis, hypothyroidism, hidradenitis suppurativa and hypotrichosis [7]. In our case study, there were no associated findings. 

The pathogenesis of these lesions has been extensively studied. Steatocystomas were initially thought to be sebaceous or retention cysts [8]. Regarding pathogenesis of this condition, Kligman and Kirchbaum [3] postulated that pluripotential ectodermal cells retain the embryonic capacity to form appendages or naevi rather than retention or inclusion cyst. Steatocystomas are now recognized as a nevoid or hamartomatous malformation of the pilosebaceous junction [1].

Eruptive vellus hair cysts can mimic steatocystoma multiplex clinically. Epidermal inclusion cysts also have to be excluded [1]. Biopsy needs to be done to exclude the differential diagnosis. The cyst wall of steatocystoma multiplex is composed of stratified squamous epithelium with the absence of granular layer. Sebaceous glands are located in the cyst wall. There is an eosinophilic cuticle on the luminal side of this wall with keratin, oil, and hairs in the lumen [1].

The definite treatment of individual lesions is excision. However, the sheer number of cysts and calcification usually precludes this type of treatment. In such instances, incision and complete expression of cysts contents or aspiration may be effective temporally in reducing the lesion [9]. Laser incision of the cyst may also be effective. Intralesional steroid, cryotherapy, and oral retinoids can be beneficial in early lesions [1]. However, eventual recurrence is the rule. We followed up the case after successful surgical excision without recurrence within 2 years.

## Figures and Tables

**Figure 3:**